# Broad Neutralization Responses Against Oncogenic Human Papillomaviruses Induced by a Minor Capsid L2 Polytope Genetically Incorporated Into Bacterial Ferritin Nanoparticles

**DOI:** 10.3389/fimmu.2020.606569

**Published:** 2020-12-04

**Authors:** Fan Yang, Filipe C. Mariz, Xueer Zhao, Gloria Spagnoli, Simone Ottonello, Martin Müller

**Affiliations:** ^1^ Research Group Tumorvirus-Specific Vaccination Strategies, Research Program Infection Inflammation & Cancer, German Cancer Research Center, Heidelberg, Germany; ^2^ Department of Chemical Life Sciences and Environmental Sustainability, University of Parma, Parma, Italy

**Keywords:** nanoparticle, human papillomavirus, ferritin, thioredoxin, neutralizing Abs, cervical cancer

## Abstract

Cervical cancer remains a global health burden despite the introduction of highly effective vaccines for the prophylaxis of causative human papillomavirus infection (HPV). Current efforts to eradicate cervical cancer focus on the development of broadly protective, cost-effective approaches. HPV minor capsid protein L2 is being recognized as a promising alternative to the major capsid protein L1 because of its ability to induce responses against a wider range of different HPV types. However, a major limitation of L2 as a source of cross-neutralizing epitopes is its lower immunogenicity compared to L1 when assembled into VLPs. Various approaches have been proposed to overcome this limitation, we developed and tested ferritin-based bio-nanoparticles displaying tandemly repeated L2 epitopes from eight different HPV types grafted onto the surface of *Pyrococcus furiosus* thioredoxin (Pf Trx). Genetic fusion of the Pf Trx-L2(8x) module to *P. furiosus* ferritin (Pf Fe) did not interfere with ferritin self-assembly into an octahedral structure composed by 24 protomers. In guinea pigs and mice, the ferritin super-scaffolded, L2 antigen induced a broadly neutralizing antibody response covering 14 oncogenic and two non-oncogenic HPV types. Immune-responsiveness lasted for at least one year and the resulting antibodies also conferred protection in a cervico-vaginal mouse model of HPV infection. Given the broad organism distribution of thioredoxin and ferritin, we also verified the lack of cross-reactivity of the antibodies elicited against the scaffolds with human thioredoxin or ferritin. Altogether, the results of this study point to *P. furiosus* ferritin nanoparticles as a robust platform for the construction of peptide-epitope-based HPV vaccines.

## Introduction

Cervical cancer results in about 270,000 mortalities worldwide annually (1, 2). Even with a cervical cytology screening system in place and with the availability of prophylactic vaccines, cervical cancer incidence and mortality are expected to increase significantly until 2030 (3). Human papillomavirus (HPV) is considered as a necessary cause for appearance of cervical lesions and further progression to established carcinoma of the cervix, as virtually all these tumors harbor single or multiple HPV infections (4–6). More than two hundred genotypes of HPV have been identified (7) and classified as mucosal or cutaneous types according to their tissue tropism. Although mucosal infections by HPV are mainly transient and spontaneously cleared by the immune system (8), persistent infections are considered major risk-factor for tumor development.

The currently licensed vaccines are based on virus-like particles (VLPs) assembled by the major capsid protein L1. VLPs are highly immunogenic and induce high titers of neutralizing antibodies, which, however, act in a pronounced HPV type-restricted manner. The reason for this is the low conservation of the neutralizing epitopes located on the surface of VLPs, despite the fact that the L1 protein is overall highly conserved among different HPV types. Moreover, L1-based VLPs have a very limited thermal stability, which makes cold-chain distribution mandatory for their preservation, a requirement that substantially constrains their potential for establishment in low- and middle-income countries where nearly 90% of the deaths by cervical cancer occur (9). Efforts to overcome these two limitations of current HPV vaccines have een undertaken. One approach for inducing broadly cross-reactive HPV-specific antibodies is to target a highly conserved neutralizing epitope located in the amino terminus of the minor capsid protein L2. In animal models, it has been shown that L2-based antigens comprising this region of the L2 protein can induce broad protection against different HPV types (8, 10–13), despite an overall low immunogenicity (14, 15). Different strategies have been proposed to improve L2 immunogenicity—e.g. displaying the L2 epitope on the surface of either HPV16 VLPs, MS2 phage capsid or adeno-associated virus particles (16–18). We have employed *Pyrococcus furiosus* thioredoxin (Pf Trx) as a scaffold for L2 epitopes presentation. This strategy allowed the generation of highly thermostable antigens capable of inducing cross-neutralizing antibody responses to multiple HPV types (12, 13, 19–23). A major step in Pf TrxL2 antigen development has been the construction of a Pf Trx-displayed polytope comprising tandemly repeated L2 epitopes from seven different mucosal oncogenic and one cutaneous HPV types. This was followed by genetic fusion of the PfTrx-L2(8x) (Pf Trx8mer) polytope with OVX313, a self-assembling polypeptide that promoted covalent heptamerization of the antigen into a super-scaffolded form, leading to a 5- to 10-fold increase in neutralizing antibody titers compared to the corresponding monomeric antigen (24, 25). The resulting nanoparticle vaccine afforded protection against 26 mucosal and cutaneous HPV types.

Due to their repetitive geometry, which allows for the ordered display of a multimeric array of immuno-epitopes, overall size and shape (i.e., density of exposed epitopes), bio-nanoparticle-based vaccines are being increasingly evaluated as virus-like particle mimics (26, 27). Indeed, the superiority of multivalent vaccine formulations, based on either natural or surrogate VLPs, with regard to the induction of higher titer and longer-lasting antibodies has been documented in various (pre) clinical settings. This has been ascribed to a highly efficient uptake into the lymphatic system and an improved cross-linking of B-cell receptors due to the higher density and valency of the antigens (28).

A particularly robust and well-developed “super-scaffold” is ferritin, which spontaneously assembles into octahedral nanoparticles (~12 nm outer diameter; molecular mass 450–500 kDa) composed by 24 subunits organized as eight trimers (4-3-2 symmetry). Genetic and, more recently, click chemistry-based ferritin-immuno-epitope fusions have been generated and tested as innovative recombinant antigens for both prophylactic and immuno-therapeutic applications (29–36). Most of these ferritin fusion antigens exploit the N-terminal three fold-axis arrangement of the ferritin subunits to reproduce a three-dimensional organization of the displayed immuno-epitopes resembling the arrangement present in the natural antigen. It was thus of interest to find out whether a similar immunogenicity and response longevity enhancement effect might also occur with a linear, relatively short epitope such as the aa. 20–38 peptide from minor capsid protein L2, which is not organized in a trimeric form on the surface of HPV virions. An additional point of interest was to ask whether higher-order multimerization, i.e., an increase in valency from seven [as in our previous, OVX-313-based nanoparticle vaccine (24, 25)] to 24 surface-exposed epitopes might further enhance the immune-performance of our HPV Trx-L2 vaccine.

We addressed these questions by generating and testing a set of nanoparticle HPV-L2 antigens of increasing complexity using *P. furiosus* ferritin (Pf Fe) as a super-scaffold. Although a clear superiority to the previous heptameric vaccine was not apparent, we found that the Pf Fe-based antigens induced robust neutralizing antibody responses against multiple HPV types both *in vitro* and *in vivo*, with a range of protection much broader than that of the licensed L1-VLP vaccines.

## Materials and Methods

### Study Design

The goal of this study was to test the immunogenicity, thermal stability and immune-performance of HPV L2-derived peptide epitopes internally fused to thioredoxin and displayed on the surface of ferritin nanoparticles. L2-beraing ferritin nanoparticles were produced and characterized biophysically and biochemically to confirm the structural integrity of the antigen. A combination of *in vitro* (PBNA) and *in vivo* (cervico-vaginal challenge mice model) studies was carried out to evaluate the immunogenicity of the nanoparticle vaccine in different pre-clinical settings and models of immunization/infection. Cross-reaction with human ferritin and thioredoxin was also evaluated.

### Plasmid Construction

Genes encoding for ferritin fusion proteins were generated by PCR and inserted into the pFBDM baculovirus transfer vector. For details of the encoded proteins refer to **Supplementary File 1**. Genes encoding for Trx-L2-OVX313 were described previously (24) and inserted into the bacterial expression vector pET34a. The L2 8mer polytope comprises the L2 epitope corresponding to HPV16 L2 aa 20–38 of HPV types 16, 18, 31, 33, 35, 6, 51, and 59. Ferritin and Trx fusion proteins are based on pGEX vectors, expression was performed in *Escherichia coli*. All of constructs were confirmed by DNA sequencing.

### Generation of Recombinant Bacmid

The recombinant donor plasmids based on pFBDM were transformed into *E. coli* DH10Bac competent cells which contained the bacmid with a mini-attTn7 target site and the helper plasmid for the site-specific transposition of nanoparticle genes from the donor vector to a bacmid DNA through lacZ gene description. The transformed cells were plated onto LB agar containing kanamycin (50 µg/ml), gentamycin (7 µg/ml), tetracycline (10 µg/ml), X-gal (100 µl/ml) and incubated at 37°C for 48 h in the presence of X-gal and IPTG. In the presence of X-gal and IPTG, the resultant recombinant bacmid produced white colonies. High molecular weight bacmids were isolated from the overnight cultures by alkaline lysis.

### Cell line and Culture Media

*Spodoptra frugipedra* (Sf9) insect cells (Sf9; Invitrogen) were grown in TNM-FH Media (Lonza, Walkersville, MD) supplemented with 10% fetal bovine serum (FBS) and 50 µg/ml penicillin and 50 µg/ml streptomycin (P/S) at 27°C and 150 rpm agitation. *Trichoplusia ni* (TN) High Five cells (Invitrogen) were routinely sub-cultured to 0.5 × 10^6^ cells/ml every 3 days in Ex-Cell™ 405 serum free medium (Sigma, SLBW9342) containing P/S at 27°C and 150 rpm agitation in 500 ml shake flasks (10–20% working volume-w/v) at 27°C and 150 rpm in the incubator. The HeLa T cell line and 293TT cells were cultured in Dulbecco’s modified Eagle medium (DMEM) supplemented with 10% FBS and P/S. The pgsa-745 cells and CHOΔfurin cells were cultured in DMEM supplemented with 10% FBS and 10 mM proline. MCF10A cells were cultured in DMEM/F12 medium supplemented with 5% horse serum, P/S, 500 µg hydrocortisone (Sigma H-0888), 10 µg/ml insulin (Sigma, I-1882), and 20 µg/ml epidermal growth factor (100-15; Peprotech).

### Antigen Purification

Nanoparticle antigens were produced in TN-High Five insect cells at 29°C in 2,000 ml shake flasks (10% w/v). Briefly, shake cultures were infected with different baculovirus at MOI 2 - 5 when the cell culture reached the concentration of 2 × 10^6^ cells/ml. After 3 days of infection, the cells were harvested and processed using two different strategies according to the construct. For Pf Fe, Pf FeTrx3mer and Pf Fe3mer, the cell cultures were cleared by centrifugation at 1,900 rpm, 30 min at 4°C. The cell pellets were resuspended in ice-cold extraction buffer (containing 5 mM MgCl_2_, 5 mM CaCl_2_, 150 mM NaCl, 0.5 mM PMSF, 0.01% (v/v) Triton X100, and 20mM HEPES in pH 7.4) and sonicated three times for 30 seconds at 3 min interval with 50% power of the ultrasonicator and cleared by centrifugation. Cell lysates were heated in to 65°C for 30 min, following centrifugation for 1 h at 12,000 × g at 4°C. Clear lysates were loaded onto a two steps gradient with 14 ml of 40% (w/v) sucrose on top of 8 ml of 71% (w/v) CsCl solution, centrifuged for 3 h at 96,500 × g at 10°C in a Beckman SW28 rotor. The interphase was collected and transferred into a 13 ml Quick-seal tube (Beckman). A CsCl gradient was produced by 16 h centrifugation at 184,000 × g at 20°C in a Sorval TFT 65.13 rotor and 1ml fraction were collected. The purity of the collected fractions was tested by SDS-PAGE. The peak fractions were pooled, dialyzed against PBS and cleared by centrifugation at 20,000 × g at 4°C for 10 min. For Pf FeTrx8mer and Pf Fe8mer, treatment of the cell lysate at 65°C for 30 min was sufficient for purification.

### Ferritin Nanoparticles Physico-Chemical Characterization

Nanoparticle formation was verified by transmission electron microscopy (TEM). Nanoparticle preparations were applied to carbon-coated grids and incubated at room temperature for 15 min. The grids were washed 3 times with PBS and stained with 1.5% uranyl acetate and then observed at × 80,000 nominal magnification with an electron microscope Zeiss EM912 and EM910. For the immune TEM, the nanoparticles were spread onto the surface of carbon-coated grids and incubated at room temperature for 15 min, then blocked with 1% BSA for 5 min. After washing the grid 3 times with PBS, mouse anti-HPV16 L2 monoclonal antibody 16L2-K4_20-38_ (12) was loaded on the grids for 1 h at room temperature, following 3 times washing with PBS 1X. Then the grids were treated with goat anti-mouse polyclonal antibody conjugated with 10 nm gold for 1 h. Finally, the grids were washed 6 times with PBS followed by negative staining with phosphotungstic acid.

Size exclusion chromatography (SEC) was performed on a Superdex 200 Increase 10/300 GL column (Lot, 10246672; GE Healthcare) equilibrated with PBS at a flow rate of 0.5 ml/min, using an Äkta fast protein liquid chromatography (FPLC) system (UV 280 nm). 1.5 MPa was set as the maximum pressure, carried out with Unicorn 5.0 (Amersham, GE Healthcare, United Kingdom). All elutions were carried out with 2 column volumes of running buffer. Fractions of 1ml were collected and stored at 4°C to be further analyzed for the protein content and composition with the Coomassie dye-binding assay and SDS-PAGE. The hydrodynamic sizes of nanoparticle antigens were analyzed by dynamic light scattering (DLS) with a Zetasizer Nano ZM (Malvern instruments, Ltd., UK).

Far-UV circular dichroism (CD) spectroscopy (190-260 nm), performed with a Jasco J715 Spectropolarimeter equipped with a Peltier temperature controller, was used to assess thermal stability of Pf FeTrx8mer dissolved in 0.1X PBS at a 1 µM concentration. CD spectra, acquired with the use of a 0.1 cm path length cuvette, a bandwidth of 1 nm, a data pitch of 0.5 nm, and a response time of 4 s were averaged from four scans. Following baseline correction, the measured ellipticity, h (mdeg), was converted to molar mean residue ellipticity [θ] (deg.cm^2^.dmol^-^), using [θ] 5h/10 cn_res_l, where θ is ellipticity, c is the molar concentration of the protein, n_res_ is the number of amino acid residues in the protein and l is the optical path length in centimeters. Thermal unfolding studies were performed in the 25–100°C temperature range, keeping the protein for 10 min at each temperature (25–30–40–50–60–70–80–90–100°C) before spectra recording. Complete protein denaturation was ensured by keeping the protein sample at 100°C for increasing lengths of time (15 min, 20 min, 30 min). An apparent Tm was determined based on 222 nm ellipticity values measured at different temperatures and calculated as inflection point of a sigmoid-fitted unfolding curve.

### Mouse and Guinea Pig Immunization

Groups of six- to eight-weeks old female BALB/c mice were purchased at Charles River; Sulzfeld, Germany (Permit number: 35-9185.81/G258/16). Ten mice per group were immunized three or four times at biweekly intervals with 20µg of different de-toxified, filter-sterilized and adjuvanted antigens in 50 µl intramuscularly. The antigens were formulated with 50% (v/v) AddaVax (InivoGen) or 50 µg aluminum hydroxide (Brenntag), and 10 µg synthetic monophosphoryl lipid A (Avanti Lipids). 350- to 400 g Outbred Hartley (Crl : HA) female guinea pigs (Charles River; Sulzfeld, Germany) (Permit number: 35-9185.81/A35/12) were immunized subcutaneously four times at biweekly intervals with 30 µg of different antigens formulated with 50% (v/v) AddaVax (InivoGen) in a volume of 100 µl. Four weeks after last immunization, animals were sacrificed and blood samples were collected by cardiac puncture.

### Pseudovirion Preparation

Pseudovirions (PsVs) were prepared by co-transfection of the human fibroblast cell line 293TT with plasmids carrying humanized HPV L1 and L2 coding sequences plus a reporter plasmid, and purification was performed by iodixanol gradient ultracentrifugation according to previously described protocol (11).

### *In Vitro* Standard L1 Pseudovirion-Based Neutralization Assays

Neutralizing antibody in sera against human papillomaviruses were detected by the L1 pseudovirion-based neutralization assay (L1-PBNA) as described previously (19, 37). Briefly, 50 µl of diluted sera in Dulbecco modified Eagle medium (DMEM) were combined with 50 µl of diluted pseudovirion in DMEM and incubated at room temperature for 30 min. Next, 50 µl of HeLa T cells (2.5 × 10^5^ cells/ml) were added to the pseudovirion-antibody mixture and incubated at 37°C for 48 h in a humidified incubator under a 5% CO_2_. The amount of secreted Gaussia luciferase was determined in 10 µl of cell culture medium using the coelenterazine substrate and Gaussia glow juice (PJK, Germany), according to the instructions of the manufacturer. A microplate luminometer (Envision 2101 multi-label reader, PerkinElmer) was used to measure culture medium associated luminescence 15 min after substrate addition. The effective inhibitory concentration (EC50) was determined using the GraphPad Prism software program, and represent the serum dilution that was able to inhibit 50% of pseudovirion infectivity. EC50 values lower than 50 were considered non-neutralizing and, therefore, set to 0.1.

### *In Vitro* L2 Pseudovirion-Based Neutralization Assays

The L2 pseudovirion based neutralization assay (L2-PBNA) was performed as previously described (38). Briefly, 100 µl of MCF10A cells at concentration of 2 × 10^5^ cells/ml were plated in a 96-well plate and propagated for 24 h to prepare extracellular matrix (ECM). After the incubation, the cells were washed twice with PBS and lysed with 50 µl pre-warmed lysis buffer [PBS containing 0.5% (v/v) Triton X-100, 20 mM NH_4_OH] at 37°C for 5 min. The ECM-coated plate was washed with 100 µl PBS per well 3 times. A pseudovirion solution was prepared in conditioned medium from CHOΔfurin cells in a total volume of 120 µl/well in the presence of 5µg/ml heparin and added to the ECM-coated wells. The Pseudoviron–furin–heparin mixture was incubated overnight at 37°C overnight. The next day, the medium was removed and the wells were washed twice with PBS. Then the sera, diluted in pgsa-745 growth medium, were added to the plates and incubated at 37°C for 3 h to allow efficient antibody binding to target epitopes. Following this incubation, pgsa-745 cells were added to the antibody containing wells to a final concentration of 1.6 × 10^5^ cells/ml. Infection was allowed to continue for 2 days at 37°C. The amount of secreted Gaussia luciferase was determined as described above.

The effective inhibitory concentration (EC50) was determined using the GraphPad Prism software program, and comprehend the serum dilution that was able to inhibit 50% of pseudovirion infection. EC50 values lower than 50 were considered non-neutralizing and, therefore, set to 0.1. All L2-PBNA related cell lines (MCF10A, CHOΔfurin, pgsa-745) were kindly provided by John Schiller’s laboratory (NIH, Bethesda, MD), and authenticity was confirmed periodically by multiplex cell authentication (MCA) assay (39).

### Cervico-Vaginal Mouse Challenge Model

The mouse cervico-vaginal challenge model was carried out as described previously (animal permit number: 35-9185.81/G128/16-01) (38). On day one, the bedding of BALB/c male mice was transferred to the cages of female mice to induce hormonal synchronization. On day three, the female mice were subcutaneously injected with 100 µl of 30 µg/µl medroxypreogesterone acetate (Depo-Provera; Pharmacia). On day five, 100 µl sera (diluted 1:2 with PBS) from immunized mice were transferred to the female mice intraperitoneally. Then the mice were treated intravaginally with 50 µl of 4% Nonoxynol-9 (N9; Spectrum) in 4% carboxymethyl cellulose (Sigma). HPV pseudoviruses encapsidating a firefly luciferase plasmid were diluted in 4% carboxymethyl cellulose (Sigma) and then instilled intra-vaginally. On day eight, Luminescence-based imaging using a Xenogen *in vivo* imaging system (IVIS) imager (Xenogen Corporation; PerkinEler) was performed. Twenty µl of luciferin substrate (15 mg/ml; Promega) were instilled intravaginally, the efficiency of protection of the antibody was assessed by *in vivo* imaging. A region of interest (ROI) analysis was performed using living image 2.50.1 software (Xenogen; PerkinElmer). Background signal was obtained and subtracted by imaging each group of mice prior to installation of luciferin.

### Pf ferritin, Human Ferritin, Pf Trx, and Human Trx Multiplex Serology

Mouse and guinea pigs’ sera were analyzed in a final dilution 1:500. Antibodies against Pf ferritin, human ferritin, Pf thioredoxin and human thioredoxin were detected using GST tagged Pf ferritin, human ferritin, Pf thioredoxin and human thioredoxin Multiplex serology as described before (40). Recombinantly GST-tagged Pf ferritin, human ferritin, Pf thioredoxin, and human thioredoxin were loaded onto glutathione-casein coupled spectrally distinct fluorescence-labeled polystyrene beads (SeroMap, Luminex Corp., Austin, TX, USA). Antibodies in sera binding to the beads were detected with streptavidin-R-Phycoerythrin labeled anti-mouse secondary antibody. The levels of antibody for each serum sample are expressed as median fluorescence intensity (MFI) of at least 100 beads per set measured, calculated by subtracting the background fluorescence as determined by a negative control.

### Ethics Approval

Animal experimentation procedures were approved by the Regierungspräsidium Karlsruhe under permit (35-9185.81/: A35/12; G258/16; G128/16-01) and were performed in accordance with the relevant guidelines and regulations. Mice and guinea pigs were kept and handled in the animal house facility of the German Cancer Research Center (DKFZ, Heidelberg) under specific pathogen-free conditions, in compliance with the regulations of the Germany Animal Protection Law.

### Statistical Analysis

Statistical significance analysis was performed with the nonparametric Mann-Whitney test performed with GraphPad Prism 8.0 (GraphPad Software, USA); differences between groups were considered significant at a P value of < 0.05.

## Results

**Figure 1:**
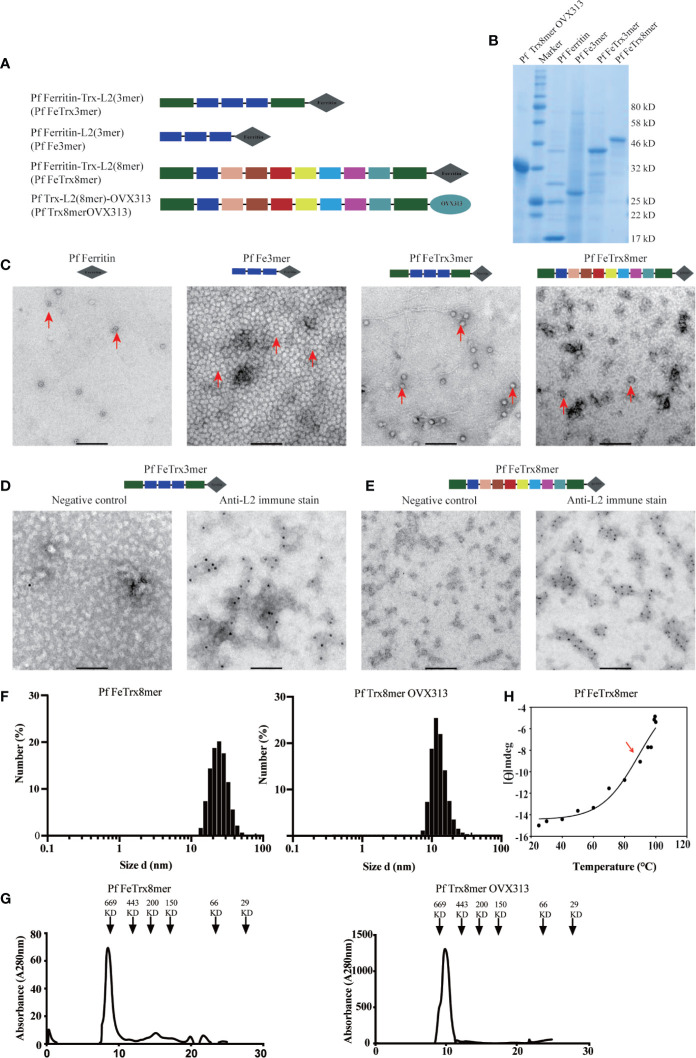
Molecular design and characterization of L2-based nanoparticle antigens. **(A)** different copies of HPV16 L2(20-38) or HPV (16, 18, 31, 33, 35, 6, 51, and 59) L2 (20-38) were fused to the amino terminus of ferritin, with or without Pf Trx, respectively. The L2 (aa20-38) of different HPVs are shown in different color according to their order. **(B)** Purified NP antigens analyzed by SDS-PAGE, **(C)**, Negative-stain transmission electron microscopy (EM) images of L2-based NP antigens. **(D, E)** immune EM of NP antigen Pf FeTrx3mer and Pf FeTrx8mer using an anti-HPV16 L2 antibody, left panels show the negative control (no primary antibody); **(F)** Dynamic light scattering analysis of the L2-based NP antigens. **(G)** Size exclusion chromatography profiles of L2-based NP antigens. **(H)** Thermal stability analysis of Pf-Fe-Trx L2(8mer) determined by circular dichroism spectroscopy. Data are presented as variations of molar ellipticity [θ] measured at 222 nm during thermal unfolding (25–100°C) of the protein. A temperature-dependent midpoint transition at 89°C was calculated by fitting the data to a sigmoid curve (see ***Methods*** section for details).

*P. furiosus* ferritin (Pf Fe) self-assembles into highly thermostable (41, 42), spherically shaped octahedral nanoparticles each composed by 24 protomers, with the N-termini of ferritin exposed on the surface of the nanoparticle. L2 is the minor capsid protein of HPV, and we have previously shown that an N-terminal region comprising amino acids 20-38 can induce broadly cross-neutralizing antibody responses when inserted into, and displayed on, the surface of *P. furiosus* thioredoxin (Pf Trx) (12, 19, 37). We have also documented a sizeable increase in immunogenicity upon fusion of Pf Trx-L2 to a self-assembling protein module (named OVX313) derived from the complement-binding protein C4bp and conversion of the antigen into a heptameric nanoparticle form (24, 25, 43). Therefore, we asked whether further multimerization of the basic Pf TrxL2 antigen would promote an additional increase in immunogenicity. To answer this question, we produced a series of PfTrxL2 super-scaffolded derivatives in which the OVX313 heptamerization module was replaced by Pf Fe (**Figure 1A**). Three copies of the HPV16 L2 aa 20–38 region or the corresponding region from eight different HPVs inserted into PfTrx were fused to either OVX313 or Pf Fe (the latter constructs are designated as Pf Fe-TrxL2(3mer) (Pf FeTrx3mer) and Pf Fe-TrxL2(8mer) (Pf FeTrx8mer), respectively; **Figure 1A**). All Pf Fe-based antigens were expressed in baculovirus-infected insect cells (see *Methods* section for details and **Figure 1B** for representative results of SDS-PAGE analysis conducted on the purified proteins), whereas the corresponding antigens harboring the OVX313 module were expressed in *E. coli* and purified as described previously (14, 24, 25) Transmission electron microscopy (TEM) revealed the presence of ferritin assemblies but the use of different fusion partners was found to affect the shape of the resulting nanoparticles. This was especially apparent in the case of the lager fusion construct [Pf FeTrx8mer], which led to the appearance of aberrant structures (**Figure 1C**). Whether this reflects the intrinsic flexibility and/or lack of a stable secondary structure of the extended L2-8mer insert or a structural alteration of Pf Fe caused by this fairly large-size insert is presently not known. However, as revealed by the immuno-EM data in **Figures 1D, E**, obtained with the use of an anti-HPV 16 L2-specific monoclonal antibody, in both constructs the L2 epitopes appear to be displayed on the surface of the Pf FeTrx3mer and Pf FeTrx8mer nanoparticles. Furthermore, despite the partial structural irregularity revealed by TEM, a rather uniform size and a single-peak elution profile were observed upon dynamic light scattering (DLS) and SEC analysis (**Figures 1F, G**). Also shown in **Figure 1H** are the results of a thermal stability analysis, performed on Pf FeTrx8mer nanoparticles by circular dichroism spectroscopy, which yielded an apparent Tm of 89°C.

### Immunogenicity and Cross-Protection Capacity of the HPV16 Monotypic, PfFe-Trx-L2 Antigen

**Figure 2:**
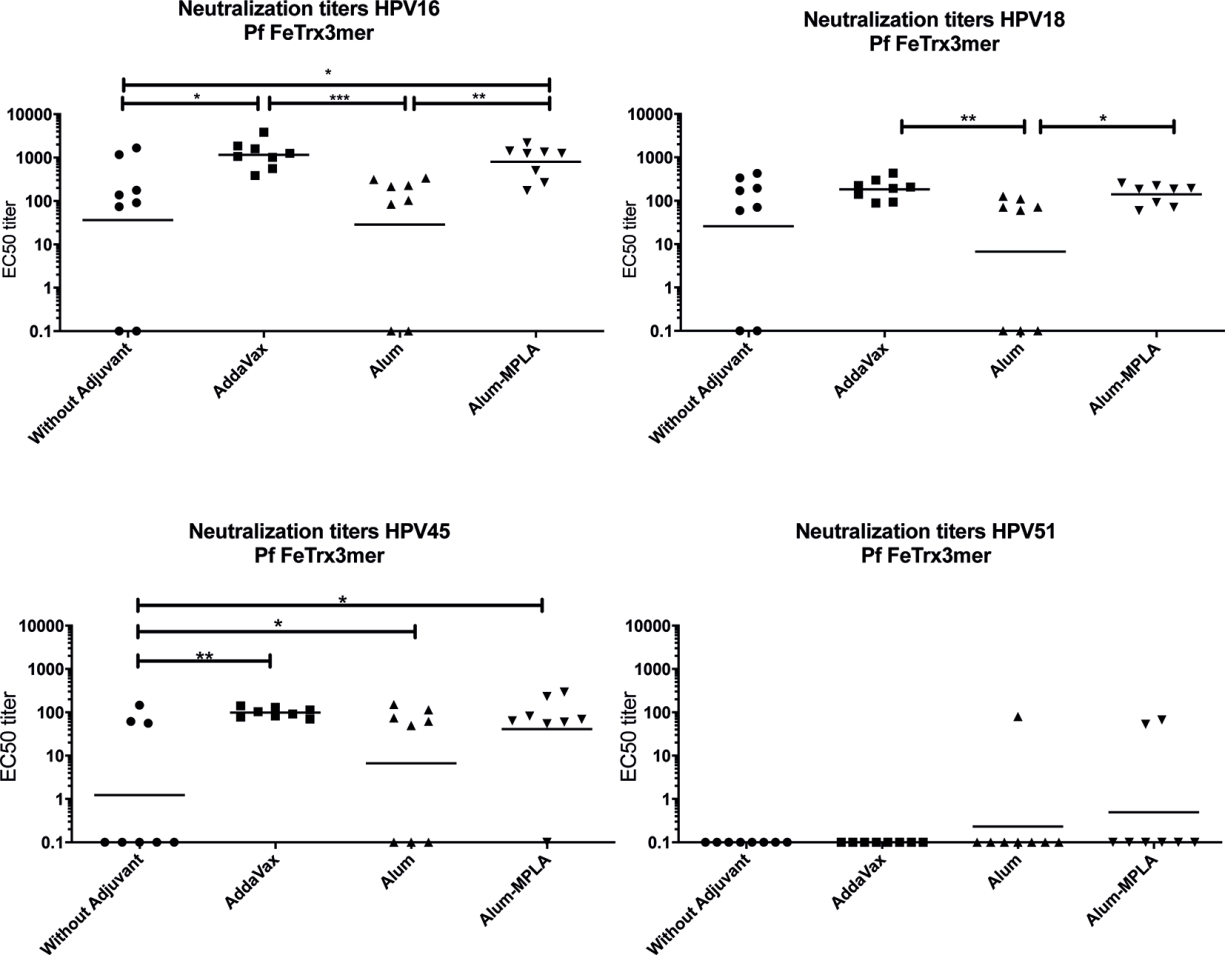
AddaVax formulation of Pf FeTrx3mer leads to superior antibody titers compared to formulation with Alum or non-adjuvanted antigen. Mice were immunized with Pf FeTrx3mer either without adjuvant or adjuvanted with AddaVax, Alum, or Alum-MPLA. Sera were analyzed by L1-based PBNA 4 weeks after the last immunization. The symbols represent the neutralizing titers of individual mice. The geometric mean values of the titers for each group are indicated by horizontal lines. A p value ≤ 0.05 was considered significant. *p < 0.05; **p < 0.01; ***p < 0.001.

Starting from Pf Fe nanoparticles harboring three copies of HPV16 L2 grafted to thioredoxin, we used three different adjuvant formulations (plus a no adjuvant control) to test the immunogenicity and cross-neutralization capacity of this antigen. To this end, neutralizing antibody titers against HPV16, 18, 31, 39, 45, and 51 were evaluated by standard (L1)-PBNA. As shown in **Figure 2**, even in absence of adjuvant, neutralizing antibodies against HPV16, 18, and 45 were induced, with titers comparable to, or higher than, those measured in the presence of alum adjuvant. The highest titers of neutralizing antibodies were achieved with Alum-MPLA- or AddaVax-adjuvanted Pf FeTrx3mer, and the latter, mono-component adjuvant was selected for all subsequent immunizations because of its ease of formulation. All four formulations induced cross-neutralizing antibodies against HPV types 18 and 45, but there was a very poor (or no) response against HPV51 (**Figure 2**), as well as against HPV 31 and 39 (data not shown). The limited cross-neutralization capacity of the antibodies generated by Pf FeTrx3mer, is in line with previous results obtained with both the basic (i.e., monovalent) and the heptameric form of the HPV-L2(3mer) antigen (24, 25).

### Grafting of the Trimeric HPV16 L2 Epitope to Thioredoxin Is Essential for Immunogenicity of the Ferritin Nanoparticle Antigen

**Figure 3:**
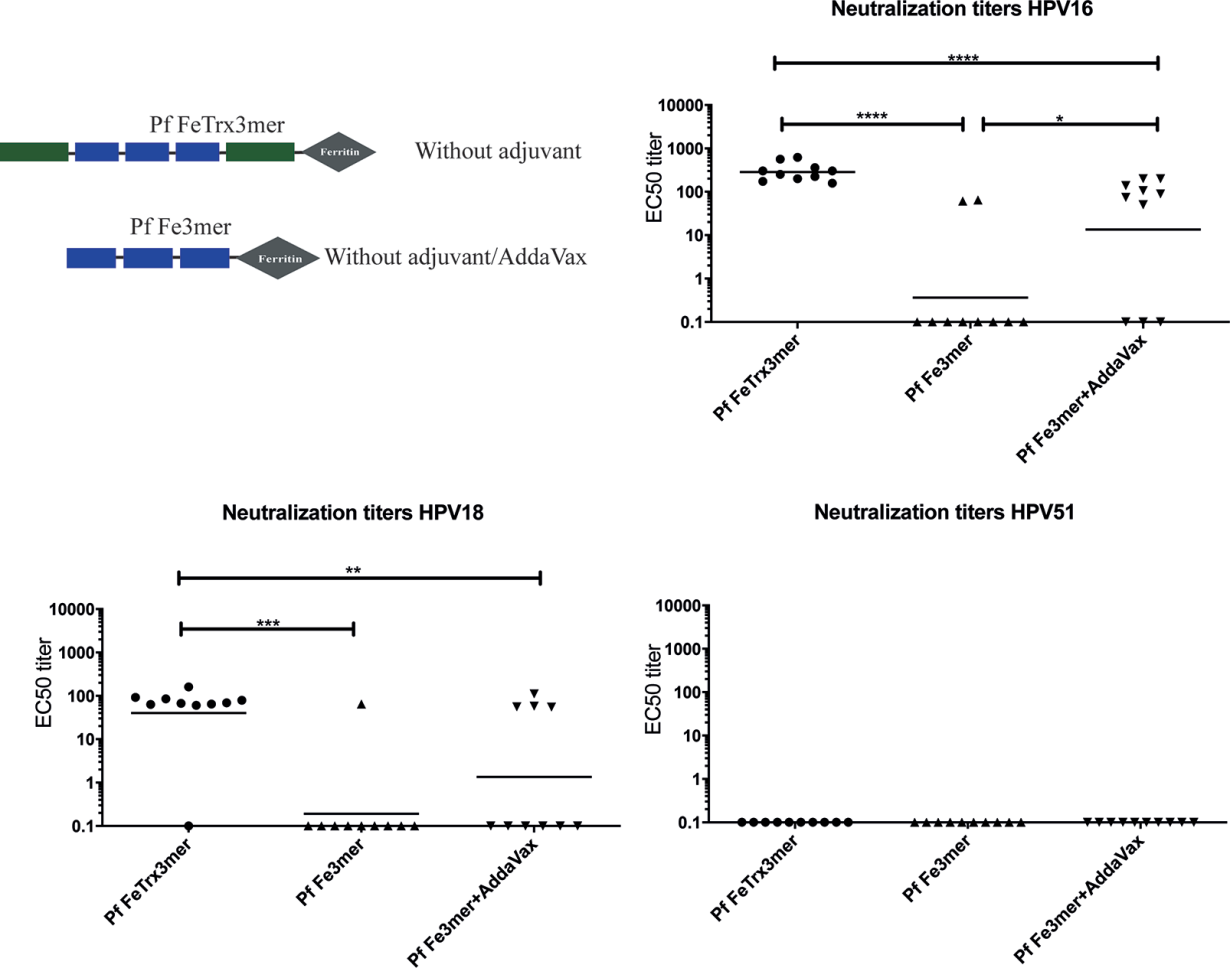
Pf Thioredoxin is indispensable in displaying L2 epitopes on Pf Ferritin nanoparticles. Mice were immunized with Pf FeTrx3mer and Pf Fe3mer, alone or adjuvanted with AddaVax four times at biweekly interval. Four weeks after the last immunization, sera were collected and analyzed by L1-PBNA. The symbols represent the neutralizing titers of individual mice. The geometric means of the titers for each group are indicated by horizontal lines. A p value of ≤ 0.05 was considered significant. *p < 0.05; **p < 0.01; ***p < 0.001; ****p < 0.00001.

Our former studies had highlighted an absolute requirement for internal grafting to, and display on the surface of, thioredoxin in order to confer high-level immunogenicity to the L2 epitopes (13, 21–23). To determine whether a similar requirement for thioredoxin also holds in the case of the ferritin-super-scaffolded nanoparticle antigen, we immunized mice with the Pf FeTrx3mer antigen and with the same antigen lacking thioredoxin (i.e., with the L2 trimer directly fused to ferritin) with and without adjuvant. As shown in **Figure 3**, the ferritin nanoparticle antigen containing the L2 epitopes grafted to thioredoxin induced significantly higher titers of neutralizing antibodies against HPV16 and 18 than the same antigen lacking thioredoxin. Interestingly, AddaVax adjuvantization did not entirely compensate for the poor immunogenicity of the Trx-lacking antigen, which yielded significantly lower antibody titers and a higher number of non-responders compared to the Trx-scaffolded antigen for both HPV16 and HPV18. Again, none of the antigen-adjuvant combinations was able to induce cross-neutralizing antibody response against HPV51 (**Figure 3**).

### Ferritin Nanoparticles Displaying A Heterotypic L2 Polytope Elicit Neutralizing Antibody Responses Against 14 High-Risk And Two Low-Risk HPV Types

**Figure 4:**
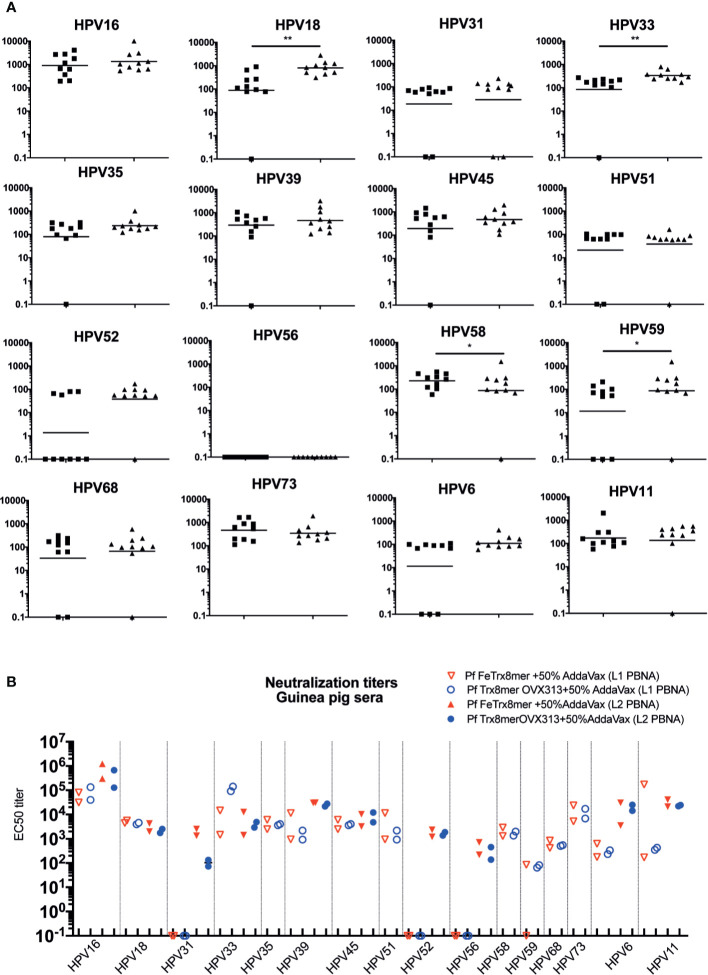
Pf FeTrx8mer adjuvanted with AddaVax induces high titers of broadly neutralizing antibodies. **(A)** Mice were immunized with AddaVax formulated Pf FTrx8mer (black squares) or Pf Trx8mer OVX313 (black triangles) antigens for four times at biweekly interval. Sera were analyzed by L1-PBNA. The geometric means of the titers for each group are indicated by horizontal lines. The y axis displays EC50 titers and a p value of ≤0.05 was considered significant. *p < 0.05; **p < 0.01. **(B)** Guinea pigs were immunized with AddaVax formulated Pf FTrx8mer or Pf Trx8mer OVX313 antigens for four times at biweekly intervals. Sera were analyzed by L1- and L2-PBNA. Symbols represent individual neutralizing titers of the animals.

Having demonstrated the basic functionality of the ferritin superscaffold, we wished to broaden the antibody response to more HPV types. Therefore, we replaced the HPV16 L2 20-38 trimer with an L2 polytope (“8mer”) harboring the neutralization epitopes from seven oncogenic HPV types plus that of the cutaneous type HPV6 (12, 24, 25). We then compared the broadness of cross-neutralization afforded by Pf FeTrx8mer with that provided by the heptameric Pf TrxL2(8mer)OVX313 (Pf Trx8mer OVX313) antigen (24, 25). Neutralizing antibody titers against 14 high-risk HPVs plus the low-risk types HPV6 and 11 were thus determined in sera from immunized mice and guinea pigs using both the standard L1-PBNA and the more sensitive L2-PBNA (see **Figures 4A, B** for mouse and guinea pig data, respectively). The titers of neutralizing antibodies induced by the two antigens against HPV 6, 16, 31, 35 and 51, all represented in the 8mer polytope, were comparable (**Figure 4A**). Slightly higher titers against HPV 18, 33, and 59, which are also represented in the polytope, were induced instead by the OVX313 nanoparticle antigen.

Cross-neutralizing antibodies against HPV types 39, 45, 52, 56, 58, 68, 73, and 11, which are all not represented in the polytope, were also determined and similar titers were measured for the two antigens (**Figure 4A**). Neither antigen induced neutralizing antibodies against HPV56 at titers detectable with the low sensitivity L1-PBNA.

A similar comparative (cross-) neutralization profiling (14 high-risk plus two low-risk HPVs) was performed in guinea pigs. As shown in **Figure 4B**, with neither antigen, antibodies against HPV 31, 52, and 56 could be detected using the low-sensitivity L1-PBNA, and only one of the two immunized animals showed a response against HPV59 after immunization with Pf FeTrx8mer. Both antigens induced neutralizing antibodies against the other 13 HPV types. As further shown in **Figure 4B**, robust (cross-) neutralization titers against HPV 31, 52, and 56 (all of which were negative in the L1-PBNA) were also detected for both antigens using the highly sensitive L2-PBNA.

### Neutralizing Antibodies Induced by Pf FeTrx8mer Are Detectable for One Year

**Figure 5:**
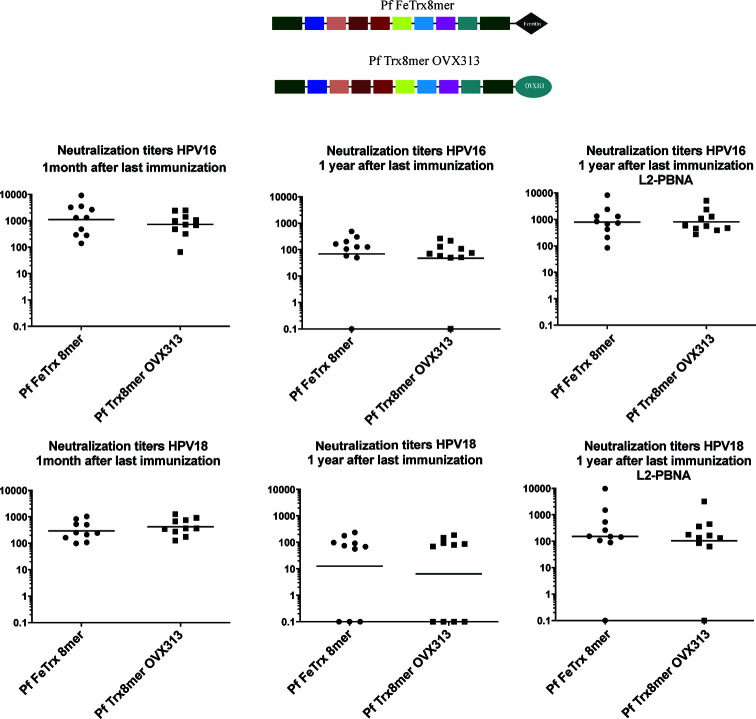
L2-based Pf ferritin nanoparticle antigen can induce long term protection against HPV16 and HPV18. Mice were immunized with Pf FeTrx8mer and Pf Trx8mer OVX313 adjuvanted with AddaVax three times biweekly. One month and one year after the third immunization, the sera were collected and for analysis by L1-PBNA, and L2-PBNA. The symbols represent the neutralizing titers of individual mice. The geometric mean titers for each group are indicated by horizontal lines.

To determine the duration of immunity induced by the two nanoparticle vaccines, mice were immunized (three times biweekly) with both antigens adjuvanted with AddaVax, followed by the assessment of antibody responses, one month and one year after the last immunization. Neutralizing antibody titers induced by Pf FeTrx8mer and Pf Trx8mer OVX313 were robust and comparable when tested one month after the last immunization (**Figure 5**). After one year, using the low sensitivity L1-PBNA, one mouse in each group showed no detectable titer against HPV16, with three and four mice negative for neutralizing anti-HPV18 antibodies in the case of Pf FeTrx8mer and Pf Trx8mer OVX313, respectively. The levels of neutralizing antibodies decreased over time: the geometric mean titers for HPV16 dropped from 1,100 to 70 (from 300 to 12 in the case of HPV18) for the Pf FeTrx8mer group and from 730 to 50 (from 420 to 10 in the case of HPV18) for the Pf Trx8mer OVX313 group. Sera from all mice were able to neutralize HPV16 when tested in the highly sensitive L2-PBNA, whereas only one mouse in each group had non-neutralizing antibody titers to HPV18.

### Neutralizing Antibodies Induced by the Pf FeTrx8mer Antigen Provide Cross-Protection in a Cervico-Vaginal Challenge Model of HPV Infection

**Figure 6:**
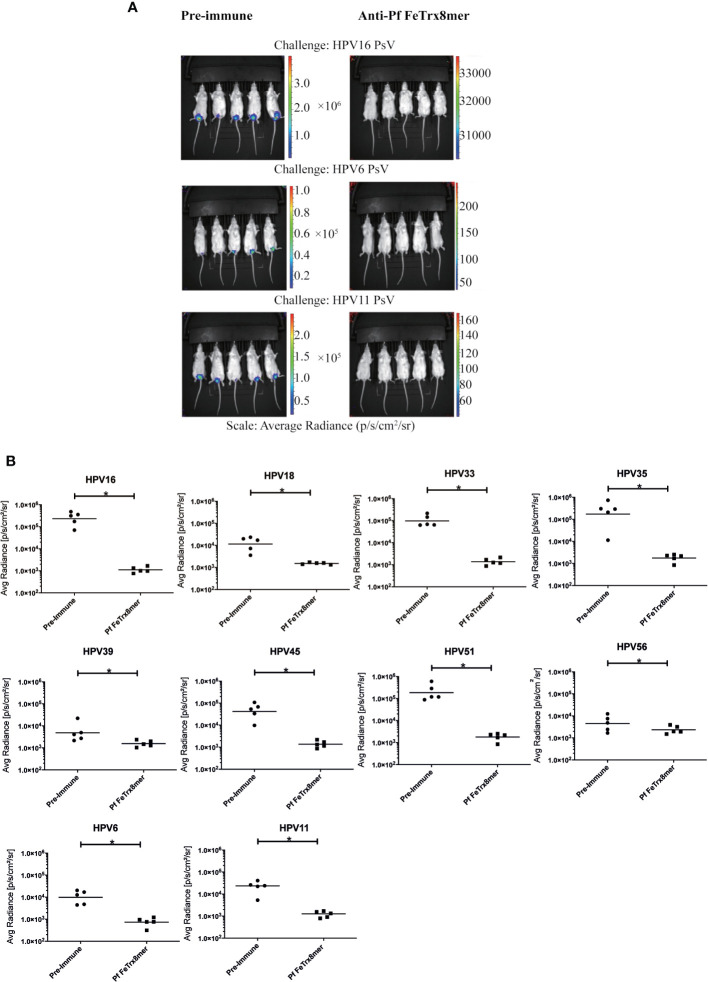
Transfer of anti-Pf FeTrx8mer sera provides protection against HPV infection *in vivo*. A pool of sera from two guinea pigs (from experiment shown in **Figure 4B**) which were immunized with the Pf FeTrx8mer was passively transferred into mice which were then challenged with HPV PSV intra-vaginally. **(A)** The pseudovirion-mediated transduction of the reporter gene was determined by *in vivo* imaging. Representative images are shown on top. The color scale shown on the right indicates the intensity of luciferase expression. **(B)** An ROI analysis for average radiance (photons per second per square centimeter per steradian) was performed with Living Image 2.50.1 software. The y axis displays the average radiance measured as photons per second per square centimeter per steradian. Due to the different *in vivo* transduction activities of the various HPV pseudovirions types, different scales were used. A p value of ≤ 0.05 was considered significant. *p ≤ 0.05.

To evaluate the *in vivo* efficacy of the Pf FeTrx8mer nanoparticle vaccine, we used a mouse challenge model of HPV infection. To this end, guinea pigs were first immunized four times with the AddaVax-adjuvanted Pf FeTrx8mer antigen. One month after the last immunization, sera were collected, pooled and transferred intraperitoneally into naïve mice, which were subsequently challenged with HPV 6, 11, 16, 18, 33, 35, 39, 45, 51, and 56 pseudovirions. *In vivo*-determined, luciferase-associated luminescence was used as indicator of the degree of infection, which was assessed two days after pseudovirion instillation; pre-immune guinea pig sera served as controls for these experiments. A representative image of vaginal infection by HPV 6, 11, 16, and 56 pseudovirion challenge is presented in **Figure 6A**. As shown in **Figure 6B**, antibodies transferred from guinea pigs immunized with the Pf FeTrx8mer antigen conferred protection against challenge with all the above HPV types.

### Earlier Immune-Response Elicited by the Pf FeTrx8mer Antigen, but Reduced Efficacy of Booster Immunization Caused by the Appearance of Anti-Ferritin Scaffold Antibodies

**Figure 7:**
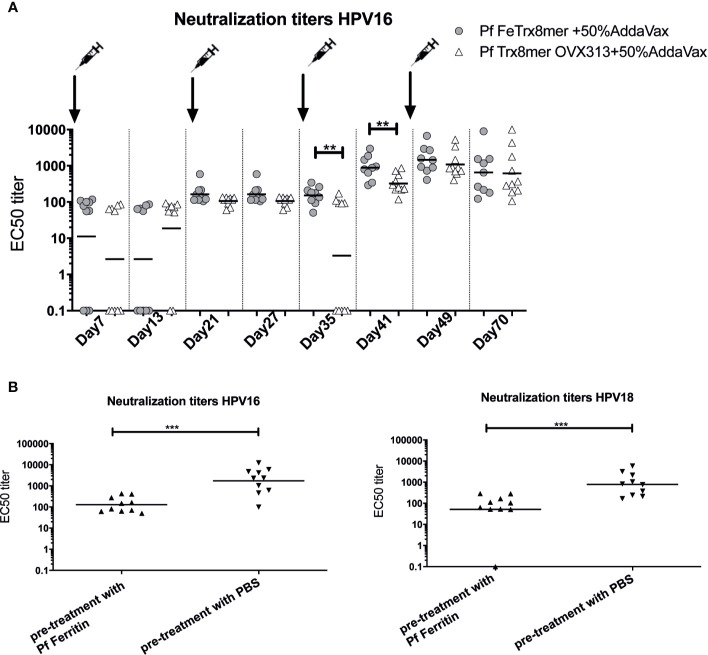
Anti-Fe scaffold antibodies interfere with induction of L2-specific neutralizing antibodies. **(A)** time course of neutralizing antibody development. Mice were immunized with Pf FeTrx8mer and Pf Trx8mer OVX313 adjuvanted with AddaVax for four times at biweekly intervals. On days 7, 13, 21, 27, 35, 41, 49, and 70 intermediate blood was collected and neutralizing antibodies determined by L1-PBNA. **(B)** Anti-L2 responses in mice after pre-immunization with empty ferritin scaffold. Mice were first immunized three times Pf ferritin, a control group was treated with PBS, followed by immunization with Pf FeTrx8mer. The antigens were adjuvanted with AddaVax. One month after the last immunization, sera were collected and analyzed by L1-PBNA. The symbols represent the neutralizing titers of individual mice. The geometric means of the titers for each group are indicated by horizontal lines. A p value of ≤0.05 was considered significant. **p < 0.01, ***p < 0.001.

To follow the development of neutralizing antibodies over time, we immunized mice four times with AddaVax-adjuvanted Pf FeTrx8mer and Pf Trx8mer OVX313 antigens. As shown in **Figure 7A**, there was a different trend in the time-course of the immune-response mounted by mice immunized with Pf FeTrx8mer compared to immunization with Pf Trx8mer OVX313. In fact, on days 35 and 41, after the third and before the fourth immunization, Pf FeTrx8mer immunized animals displayed significantly higher antibody titers compared to animals immunized in parallel with Pf Trx8mer OVX313. This difference, however, disappeared after the fourth immunization, indicating a loss of booster effect over time for the Pf FeTrx8mer antigen. We hypothesized that this could be due to the production of anti-ferritin scaffold antibodies that would then interfere with the induction of L2-specific neutralizing antibodies. To test this hypothesis, we pretreated mice with ferritin only (Pf Fe pre-immunization group) or PBS (control group) for three times biweekly followed by a standard three-dose immunization with the Pf FeTrx8mer antigen. One month after the last immunization, neutralizing antibodies against HPV16 and HPV18 were determined by the L1-PBNA. As shown in **Figure 7B**, the titers of anti-HPV16 and anti-HPV18 neutralizing antibodies in PBS-treated mice, i.e., mice without pre-existing anti-ferritin antibodies at the time of HPV vaccination were on average 19-fold higher than those of ferritin-pretreated animals. In keeping with similar results previously obtained with other ferritin nanoparticle antigens (29, 44), this indicates an interference of pre-existing anti-ferritin antibodies with the induction of HPV neutralizing responses.

### Antibodies Induced by the Pf FeTrx8mer Antigen Do Not Cross-React With Human Ferritin nor Thioredoxin

**Figure 8:**
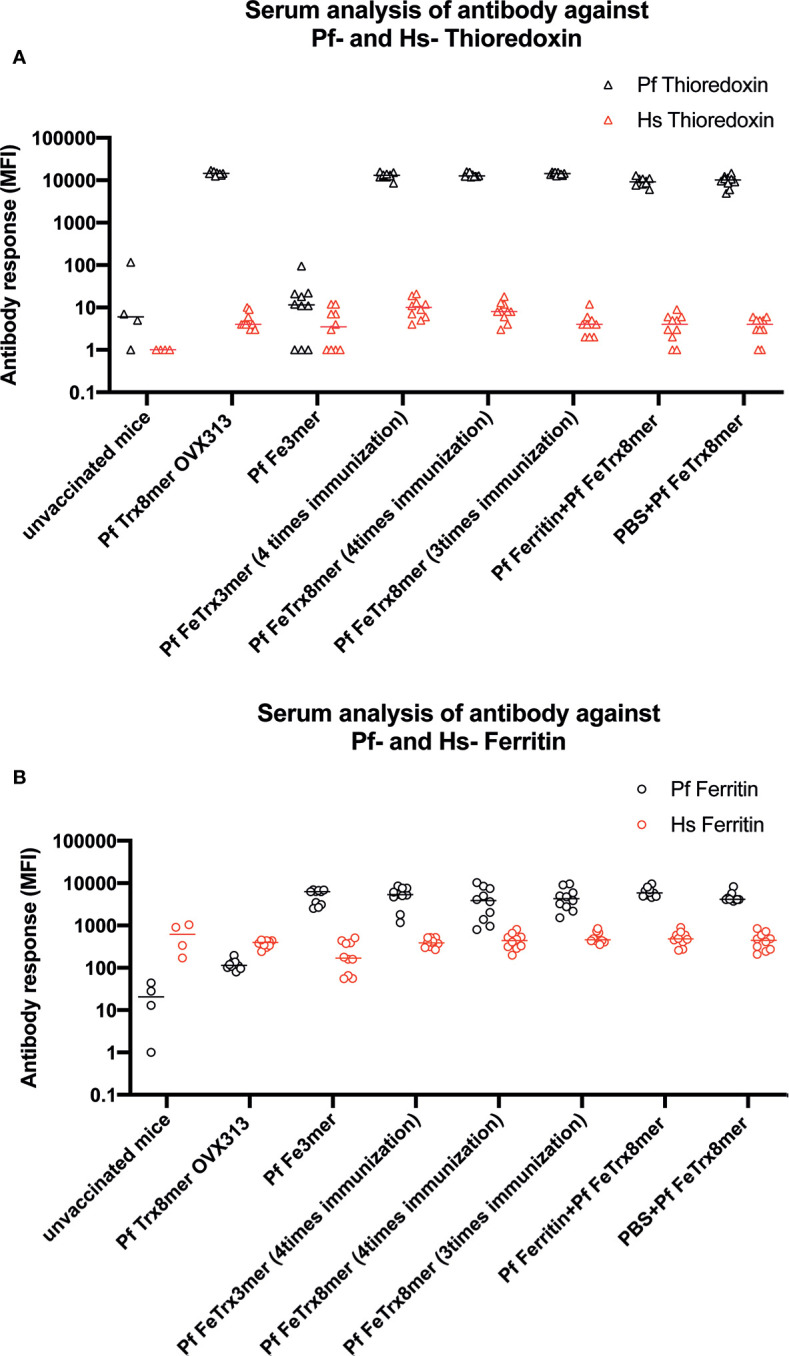
Pf Fe Trx antigens do not induce antibody responses against their human counterparts. Quantitative antibody measurements (MFI) with reference to sero status in ferritin and thioredoxin species-specific monoplex serology. **(A)** Measurement of antibodies against Pf and human ferritin. **(B)** Measurement of antibodies against Pf and human thioredoxin.

The sequence homology between *Pyrococcus* ferritin and thioredoxin and their human counterparts is 26.7 and 12.3%, respectively (**Figure S1**). To determine whether the anti-ferritin and anti-thioredoxin antibodies induced by the Pf FeTrx8mer and Pf Trx8mer OVX313 antigens cross-react with human ferritin or human thioredoxin, we employed a Luminex assay using bead-immobilized human and *P. furiosus* proteins fused to GST. A total of seventy sera derived from different immunization experiments were analyzed, using sera from unvaccinated or Pf Trx8mer OVX313 vaccinated mice as negative controls. As shown in **Figure 8**, all sera from mice immunized with the Pf FeTrx8mer antigen strongly reacted with both *Pyrococcus* ferritin (**Figure 8A**, MFI: 5102.8) and thioredoxin (**Figure 8B**, MFI: 12378.6), while only a background reactivity (MFI values of 21.5 and 32.5, for Pf Fe and Pf Trx, respectively) was observed with sera from unvaccinated control animals. Despite the relatively high background MFI values measured in the assay employing human ferritin as capture antigen, there was no cross-reactivity of these sera with the homologous human proteins: MFI values of 432.5 and 6.2 for human ferritin and thioredoxin, respectively, compared to an MFI value of 614.25 for the negative controls. This indicates that immunization with Pf FeTrx8mer leads to strong responses against the *Pyrococcus* thioredoxin and ferritin scaffolds but the induced antibodies do not recognize the corresponding human proteins.

## Discussion

Natural infection with HPV induces neutralizing antibody responses, yet geometric mean titers (GMT) are usually low (anti-HPV16: 14.69 EU/ml, anti-HPV18: 14.66 EU/ml) (45). These responses are directed against the L1 protein, and there are no reports of anti-L2 neutralizing antibodies after natural infection with HPV. Unlike the commercial L1-VLP vaccines, the neutralization epitopes of the minor capsid protein L2 induce a broadly protective immune response. As neither L2 nor the major cross-neutralization epitope located in the amino terminus of L2 assembles into higher-order structures, L2 epitopes need to be presented on a protein carrier acting as a macromolecular scaffold (46).

Antigen presentation *via* ferritin nanoparticles (NP) has proven highly potent at improving induction of humoral immune responses (47–49). Therefore, in this study, we have engineered minor capsid protein L2 epitopes genetically fused to Pf ferritin, and comprehensively investigated the immunogenicity of the resulting NP antigens. Pf ferritin NPs displaying an L2-derived polytope on their surface induced robust neutralizing antibody titers against 14 high-risk and two low-risk mucosal HPV types. The resulting antibodies provided protection in a cervico-vaginal mouse model of HPV infection. Of note, the immune-response elicited by the Pf Fe-Trx-L2 antigen, which displayed a certain degree of self-adjuvancy and a strong requirement for the primary Pf Trx scaffold, was also characterized by an early appearance of long-lasting antibody titers. Our data also shows a requirement to present the L2 polytope in the context of Trx, even when using the ferritin scaffold (**Figure 3**). In a previous publication, we also observed that multimerization increases immunogenicity of L2 polytopes but that presentation of the epitopes in context of thioredoxin is still required (25). Presenting L2 epitopes on a KLH scaffold resulted in virtually no immunogenicity in respect to induction of neutralizing antibodies, although KHL itself is highly immunogenic in BALB/c mice (21). Thus, this indicates that in fact the thioredoxin scaffold is important for L2 presentation and does not “only” provide T-helper epitopes. This is also in line with our recently reported findings demonstrating the pivotal role of the Pf Trx scaffold for efficient induction of not only Th2 but also Th1 immune responses (50). A possible drawback, likely due to the strong immunogenicity of *Pyrococcus* ferritin, was the induction of anti-scaffold antibodies that may interfere with the establishment of a fully effective anti-HPV L2 neutralizing response. We observed that pre-vaccination with ferritin significantly reduced the anti-L2 response against the Pf Fe Trx8mer antigen which could explain why the third booster immunization did not yield higher neutralizing titers. However, we cannot exclude the possibility that the lack of this booster effect is because anti-L2 antibody titers have already reached a plateau. Similar off-target effects have been reported previously for other ferritin (and other super-scaffold) based nanoparticle antigens (29, 30, 51). Importantly, however, no antibodies cross-reactive with the human counterparts of *Pyrococcus* thioredoxin and ferritin were elicited by the Pf Fe-Trx-L2 antigen.

Current HPV vaccines represent extremely powerful strategies for preventing HPV-induced tumors. However, only 91 countries have included the HPV vaccines in their national immunization programs, which means that in more than half of the world (especially low-resource areas) an adequate vaccine coverage for HPV-related cancers and other pathologies is missing (52). Here, the cost and the reduced thermal stability of the current vaccines are major factors limiting their distribution. Moreover, the VLP vaccines are highly type specific, they fail to induce protection against all high-risk HPV types, although this gap has been reduced with the introduction of the nonavalent Gardasil 9 vaccine (53).

Antigen formulation with adjuvants is a strategy to boost humoral and cellular immunogenicity.

Three adjuvants were tested and compared with the non-adjuvanted antigen. The squalene-based adjuvant AddaVax elicited the strongest responses, but a well detectable, albeit weaker, response was also observed with the non-adjuvanted ferritin NP-based antigen. This self-adjuvancy may reflect the ability of ferritin nanoparticles to promote antigen depot but the additional contribution of ferritin-harbored T helper epitopes cannot be excluded (54). On the other hand, the largely sub-optimal adjuvancy observed with alum likely reflects a disruptive effect of aluminum salts upon adsorption to ferritin nanoparticles.

A major goal of the present work was the achievement of broad HPV protection. A previously developed L2 polytope antigen heptamerized by genetic fusion to an engineered C4bp domain (OVX313) (24, 25) served as reference in the present study. According to our previous data, the cross-neutralization epitope of HPV16 does not induce robust neutralizing antibodies against certain HPV types such as HPV31 and HPV51 (**Figure 2**) (12, 13, 19, 37). The use of an octameric L2 polytope covering seven different high-risk HPVs, selected on the basis of a homology analysis, plus one low-risk HPV afforded protection against 14 high-risk and two low-risk HPV types. Neutralizing antibodies against HPV31, 52 and 56 could not be detected with the highly stringent but low-sensitivity L1-PBNA. They could be readily detected, however, using the highly sensitive L2-PBNA, which more closely mimics the natural infection. Moreover, we confirmed the *in vivo* functionality of vaccine-induced neutralizing antibodies by transferring guinea pig immune sera to naïve-recipient mice and subsequent demonstration of passive immunization against the challenge with ten HPV types, including HPV56. These are important findings, since (i) *in vitro* neutralization not always correlates to *in vivo* protection, and (ii) neutralizing antibody levels are not always detectable *in vitro*. HPV vaccines are primarily recommended for adolescent girls; however, even adult women face the risk of HPV infection throughout their sexually active life. The duration of protection thus represents a critical issue for the overall, especially long-term, effectiveness of HPV prophylactic vaccines. Indeed, vaccine-induced antibodies peaked one month after the last immunization but could still be detected 11 years after administration of the current VLP vaccines (55). Memory B cells and long-lived plasma cells are key to immunization longevity and long-term protection. In long-term experiments, we detected a rapid increase in type-specific anti-HPV antibody levels, indicating that the Pf FeTrx8mer vaccine efficiently activates B cells. Neutralizing antibodies were still well detectable one year after the last immunization, albeit with decreased titers.

Safety is always a major concern in prophylactic vaccine development. Besides potential toxicity of antigen-adjuvant formulations, molecular mimicry, i.e., the induction of cross-reactive responses against endogenous host proteins is also a great concern (56–58). Our results indicate that neither Pf ferritin, nor Pf Trx induce antibodies that cross-react with the corresponding human homologs.

To develop a successful vaccine, the antigen should preferentially present repetitive B cell epitopes to activate B cells, and could be recognized by APCs and activate T helper cells to help B cells to produce antibody. As for the size and epitopes repetitive presenting, Pf FeTrx8mer should be more favorable, however, they share a similar immunogenicity, might be the central core domain of OVX313 contributes to bind to CD40 on B cells (59). In summary, we proved the NP vaccine antigen based on minor capsid protein L2 epitopes is capable of inducing cross-neutralizing antibodies against 14 oncogenic types of HPV and two non-oncogenic types of HPV.

## Data Availability Statement

The datasets presented in this study can be found in online repositories. The names of the repository/repositories and accession number(s) can be found in the article/**Supplementary Material**.

## Ethics Statement

The animal study was reviewed and approved by the Regierungspräsidium Karlsruhe under permit (35-9185.81/: A35/12; G258/16; G128/16-01).

## Author Contributions

FY and MM designed research studies. FY performed the research. FM, XZ, and FY participated in animal immunizations. GS and SO performed CD analysis. FY, SO, and MM interpreted and discussed the data. FY wrote the paper. FY, FM, SO, and MM participated in manuscript revision. All authors contributed to the article and approved the submitted version.

## Funding

FY was supported by Chinese Scholarship Council (201506160063). XZ was supported by the Wilhelm-Sander-Stiftung (2013.136.3). Co-funding of GS fellowship by the Interuniversity Consortium for Biotechnology (CIB) is also gratefully acknowledged. This work also benefited from instrumentation facilities made available within the COMP-HUB Initiative, funded by the “Departments of Excellence” program of the Italian Ministry for Education, University and Research (MIUR, 2018–2022).

## Conflict of Interest

At the time the research described in this manuscript was performed, FY, FM, XZ, and MM were employees of DKFZ, while GS and SO were employees of University of Parma.
