# Supplementary material for: Broad Neutralization Responses Against Oncogenic Human Papillomaviruses Induced by a Minor Capsid L2 Polytope Genetically Incorporated Into Bacterial Ferritin Nanoparticles
Source: Front Immunol. 2020 Dec 4;11:606569. doi: 10.3389/fimmu.2020.606569 (PMC7746619; doi:10.3389/fimmu.2020.606569)
Supplement: Supplementary file 1 [file DataSheet_1.docx]

**Supplementary Materials:**

**Data file S1.** Amino acid sequences of all nanoparticle antigen construct.

Pf Ferritin

MLSERMLKALNDQLNRELYSAYLYFAMAAYFEDLGLEGFANWMKAQAEEEIGHALRFYNYIYDRNGRVELDEIPKPPKEWESPLKAFEAAYEHEKFISKSIYELAALAEEEKDYSTRAFLEWFINEQVEEEASVKKILDKLKFAKDSPQILFMLDKELSARAPKLPGLLMQGGE

Pf FeTrx8m

MIIEYDGEIDFTKGRVVLWFSIPGCGPKTCKQAGTCPPDIIPKVEGGGPKTCKQSGTCPPDVVPKVEGGGPQTCKAAGTCPSDVIPKIEHGGPQTCKATGTCPPDVIPKVEGGGPRTCKAAGTCPPDVIPKVEGGGPQTCKLTGTCPPDVIPKVEHGGPSTCKAAGTCPPDVVNKVEGGGPKTCKQAGTCPSDVINKVEGGGPCRLVERFMTELSEYFEDIQIVHINAGKWKNIVDKFNILNVPTLVYLKDGREVGRQNLIRSKEEILKKLKELQEMESQVRQNLSERMLKALNDQLNRELYSAYLYFAMAAYFEDLGLEGFANWMKAQAEEEIGHALRFYNYIYDRNGRVELDEIPKPPKEWESPLKAFEAAYEHEKFISKSIYELAALAEEEKDYSTRAFLEWFINEQVEEEASVKKILDKLKFAKDSPQILFMLDKELSARAPKLPGLLMQGGE

Pf Fe3mer

MGGPKTCKQAGTCPPDIIPKVEGGGPKTCKQAGTCPPDIIPKVEGGGPKTCKQAGTCPPDIIPKVEGGGPMESQVRQNLSERMLKALNDQLNRELYSAYLYFAMAAYFEDLGLEGFANWMKAQAEEEIGHALRFYNYIYDRNGRVELDEIPKPPKEWESPLKAFEAAYEHEKFISKSIYELAALAEEEKDYSTRAFLEWFINEQVEEEASVKKILDKLKFAKDSPQILFMLDKELSARAPKLPGLLMQGGE

Pf FeTrx3mer

MIIEYDGEIDFTKGRVVLWFSIPGCGPKTCKQAGTCPPDIIPKVEGGGPKTCKQAGTCPPDIIPKVEGGGPKTCKQAGTCPPDIIPKVEGGGPCRLVERFMTELSEYFEDIQIVHINAGKWKNIVDKFNILNVPTLVYLKDGREVGRQNLIRSKEEILKKLKELQEMESQVRQNLSERMLKALNDQLNRELYSAYLYFAMAAYFEDLGLEGFANWMKAQAEEEIGHALRFYNYIYDRNGRVELDEIPKPPKEWESPLKAFEAAYEHEKFISKSIYELAALAEEEKDYSTRAFLEWFINEQVEEEASVKKILDKLKFAKDSPQILFMLDKELSARAPKLPGLLMQGGE

Pf Trx8mer OVX313

MIIEYDGEIDFTKGRVVLWFSIPGCGPKTCKQAGTCPPDIIPKVEGGGPKTCKQSGTCPPDVVPKVEGGGPQTCKAAGTCPSDVIPKIEHGGPQTCKATGTCPPDVIPKVEGGGPRTCKAAGTCPPDVIPKVEGGGPQTCKLTGTCPPDVIPKVEHGGPSTCKAAGTCPPDVVNKVEGGGPKTCKQAGTCPSDVINKVEGGGPCRLVERFMTELSEYFEDIQIVHINAGKWKNIVDKFNILNVPTLVYLKDGREVGRQNLIRSKEEILKKLKELQEGSKKQGDADVCGEVAYIQSVVSDCHVPTAELRTLLEIRKLFLEIQKLKVEGRRRRRS

Pf Trx-GST

MSPILGYWKIKGLVQPTRLLLEYLEEKYEEHLYERDEGDKWRNKKFELGLEFPNLPYYIDGDVKLTQSMAIIRYIADKHNMLGGCPKERAEISMLEGAVLDIRYGVSRIAYSKDFETLKVDFLSKLPEMLKMFEDRLCHKTYLNGDHVTHPDFMLYDALDVVLYMDPMCLDAFPKLVCFKKRIEAIPQIDKYLKSSKYIAWPLQGWQATFGGGDHPPKSDLVPRGSPGMIIEYDGEIDFTKGRVVLWFSIPGCGPCRLVERFMTELSEYFEDIQIVHINAGKWKNIVDKFNILNVPTLVYLKDGREVGRQNLIRSKEEILKKLKELQEVDKPPTPPPEPET

Homo Trx-GST

MSPILGYWKIKGLVQPTRLLLEYLEEKYEEHLYERDEGDKWRNKKFELGLEFPNLPYYIDGDVKLTQSMAIIRYIADKHNMLGGCPKERAEISMLEGAVLDIRYGVSRIAYSKDFETLKVDFLSKLPEMLKMFEDRLCHKTYLNGDHVTHPDFMLYDALDVVLYMDPMCLDAFPKLVCFKKRIEAIPQIDKYLKSSKYIAWPLQGWQATFGGGDHPPKSDLVPRGSPGMVKQIESKTAFQEALDAAGDKLVVVDFSATWCGPCKMIKPFFHSLSEKYSNVIFLEVDVDDCQDVASECEVKCMPTFQFFKKGQKVGEFSGANKEKLEATINELVVDKPPTPPPEPET

Pf ferritin-GST

MSPILGYWKIKGLVQPTRLLLEYLEEKYEEHLYERDEGDKWRNKKFELGLEFPNLPYYIDGDVKLTQSMAIIRYIADKHNMLGGCPKERAEISMLEGAVLDIRYGVSRIAYSKDFETLKVDFLSKLPEMLKMFEDRLCHKTYLNGDHVTHPDFMLYDALDVVLYMDPMCLDAFPKLVCFKKRIEAIPQIDKYLKSSKYIAWPLQGWQATFGGGDHPPKSDLVPRGSNWMKAQAEEEIGHALRFYNYIYDRNGRVELDEIPKPPKEWESPLKAFEAAYEHEKFISKSIYELAALAEEEKDYSTRAFLEWFINEQVEEEASVKKILDKLKFAKD

Homo Ferritin-GST

MSPILGYWKIKGLVQPTRLLLEYLEEKYEEHLYERDEGDKWRNKKFELGLEFPNLPYYIDGDVKLTQSMAIIRYIADKHNMLGGCPKERAEISMLEGAVLDIRYGVSRIAYSKDFETLKVDFLSKLPEMLKMFEDRLCHKTYLNGDHVTHPDFMLYDALDVVLYMDPMCLDAFPKLVCFKKRIEAIPQIDKYLKSSKYIAWPLQGWQATFGGGDHPPKSDLVPRPWSNQTSLYKKAERET

Fig S1.

**Fig S1. The alignment of Pf ferritin and human ferritin (A), Pf thioredoxin and Human thioredoxin (B).** * indicates the position containing the fully conserved residue; : indicates strong similarity of residue between two spices; . Indicates limited similarity of residue between two spices
